# Chemical Features and Machine Learning Assisted Predictions of Protein-Ligand Short Hydrogen Bonds

**DOI:** 10.21203/rs.3.rs-2895170/v1

**Published:** 2023-05-15

**Authors:** Shengmin Zhou, Yuanhao Liu, Sijian Wang, Lu Wang

**Affiliations:** 1 YDS Pharmatech, Inc., Albany, NY 12226, USA; 2 Department of Statistics, Institute for Quantitative Biomedicine, Rutgers University, Piscataway, NJ 08854, USA; 3 Department of Chemistry and Chemical Biology, Institute for Quantitative Biomedicine, Rutgers University, Piscataway, NJ 08854, USA

## Abstract

There are continuous efforts to elucidate the structure and biological functions of short hydrogen bonds (SHBs), whose donor and acceptor heteroatoms reside more than 0.3 Å closer than the sum of their van der Waals radii. In this work, we evaluate 1070 atomic-resolution protein structures and characterize the common chemical features of SHBs formed between the side chains of amino acids and small molecule ligands. We then develop a machine learning assisted prediction of protein-ligand SHBs (MAPSHB-Ligand) model and reveal that the types of amino acids and ligand functional groups as well as the sequence of neighboring residues are essential factors that determine the class of protein-ligand hydrogen bonds. The MAPSHB-Ligand model and its implementation on our web server enable the effective identification of protein-ligand SHBs in proteins, which will facilitate the design of biomolecules and ligands that exploit these close contacts for enhanced functions.

## Introduction

Hydrogen bonding plays essential roles in mediating the structure, conformational transformation and biological functions of proteins. Canonical hydrogen bonds form from amino acid residues and ligands that contain O or N atoms and the distances between the heteroatoms, R, usually fall within the range of 2.8 – 3.2 Å.^1^ In addition to these normal hydrogen bonds (NHBs), short hydrogen bonds (SHBs) with R ≤ 2.7 Å are often observed on the surface and in the active cavities of proteins, possibly because their three-dimensional folds can bring the polypeptide backbone, polar side chains and bound ligands into close proximity.^2–6^ As the proton donor and acceptor atoms reside more than 10% closer than the sum of their van der Waals radii, SHB interactions deviate significantly from simple electrostatic forces, and instead exhibit strong covalent characters that arise from the quantum mechanical delocalization of both the electrons and protons.^5,7–16^ For example, when R shortens, the electronic energy surface for shuttling the proton in a hydrogen bond varies gradually from a double-well potential to a single-well potential with a diminishing barrier.^6–9^ In the limit that R becomes shorter than 2.4 Å, the proton potential energy surface is essentially barrierless. In these cases, electronic and nuclear quantum effects combine to weaken the confinement of Donor-H bond and enable the proton to be shared between the donor and acceptor groups.

A notable type of SHBs is low-barrier hydrogen bonds where the proton transfer barrier is comparable to the zero-point energy of an O-H or N-H vibration, which is typically around 5 kcal/mol. It is proposed that the energy barrier becomes sufficiently low when R of a hydrogen bond lies between 2.45 and 2.65 Å and the proton affinities of the donor and acceptor groups are closely matched. In such a compact structure, nuclear quantum effects allow the proton to move freely between the heteroatoms and the hydrogen bond becomes exceptionally strong.^17–20^ Low-barrier hydrogen bonds are often observed in the active site of proteins, and hence are associated with a variety of biological processes ranging from stabilizing the reaction intermediates in enzyme catalysis to regulating the binding of antibiotics in bacterial proteins and promoting biological signal transmission.^18,20–29^ Since their original proposal,^17^ low-barrier hydrogen bonds have been under extensive investigations although their geometry, strength and functional importance are still under debate.^30–36^ Conventionally, NMR spectroscopy is widely used for their exploration because the delocalized protons exhibit characteristically downfield chemical shifts and distinct isotope effects when replaced with deuterium.^9,18–21,24,37^ More recently, advancements in X-ray and neutron diffraction and optical spectroscopy have enabled the direct detection of the position and local environment of protons, providing crucial information on the structure and behavior of low-barrier hydrogen bonds in large proteins.^23,25–29,35,36^

SHBs constitute approximately 24% of the hydrogen bonds that form between the side chains of amino acids and are frequently observed linking active-site residues and ligands.^2–6,38,39^ However, unambiguous identification of these compact structures can only be achieved when proteins are resolved at atomic resolution (≤1.2 Å), which remain a demanding task for structure determination techniques such as X-ray and neutron scattering, NMR spectroscopy and cryo-electron microscopy single particle analysis. From a computational perspective, molecular mechanics based refinement and prediction methods typically rely on classical force fields that impose strong repulsion between close-by atoms and prevent the formation of SHBs.^40–42^ As a result of the coordinate errors in the crystal structures and the inaccuracies of conventional force fields, SHBs are often overlooked in the construction and prediction of protein structures. To tackle these challenges, we have recently designed the machine learning assisted prediction of SHBs (MAPSHB) model, which uses 21 structural, chemical and sequence features to detect SHBs that connect amino acid residues in proteins.^38^ In this work, we focus on hydrogen bonds between amino acids and small molecule ligands, such as enzyme cofactors and drugs, and create the machine learning assisted prediction of protein-ligand SHBs (MAPSHB-Ligand) model based on their common chemical and sequence characteristics.

## Results and Discussion

**Figure 1.**
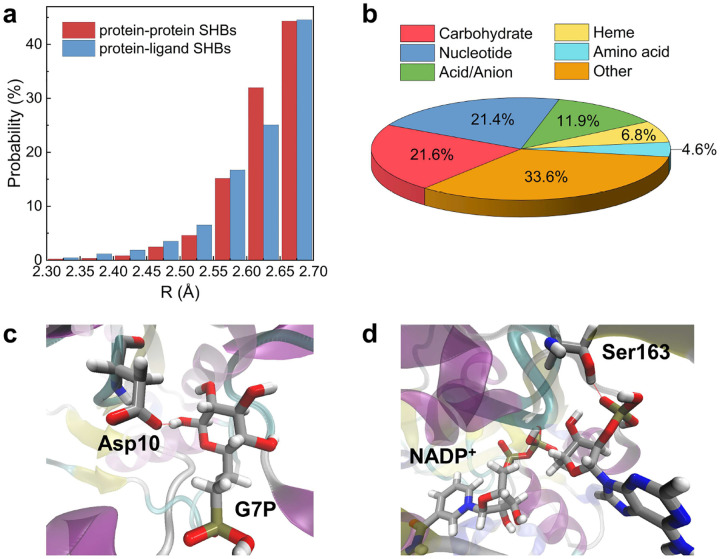
(a) Probability distributions of the 7070 protein-protein SHBs and 1272 protein-ligand SHBs at different R. (b) Distribution of ligand categories in the protein-ligand SHBs. Example structures of SHBs formed between (c) Asp10 and G7P in the active site of *β*-phosphoglucomutase (PDB ID 2WF7)^44^ and (d) Ser163 and NADP^+^ in the active site of curacin cyclopropanase (PDB ID 5DP2).^45^ In the structures, silver, red, blue, tan and white represent the C, O, N, P and H atoms, respectively.

From the Protein Data Bank (PDB),^43^ we collect 1070 crystal structures of protein-ligand complexes that are refined from X-ray or neutron diffraction experiments and have resolution greater than or equal to 1.1 Å. These atomic-resolution structures allow us to locate the O and N atoms with a coordinate error of 0.1 Å and properly distinguish SHBs from NHBs. After assessing each protein-protein and protein-ligand hydrogen bond, we categorize it as a SHB if its R is within 2.3 to 2.7 Å, or as a NHB if its R falls between 2.8 and 3.2 Å. In these analyses, we only consider the side chains of the amino acid residues given their frequent occurrence in SHBs (Tables S1 and S2), and ignore the polyol and inorganic anion ligands since they are primarily used in the preparation of protein crystals and are less likely to participate in biological functions (Table S4). This search yields 7070 SHBs and 22353 NHBs that link two amino acids, and 1272 SHBs and 2733 NHBs that involve both amino acids and small molecule ligands. Therefore, SHBs are present in approximately 1 out of every 3 protein-ligand hydrogen bonds and every 4 protein-protein hydrogen bonds, highlighting their prevalence in protein-ligand complexes. From Figure 1a, although there are fewer ligand-containing SHBs than those involving only amino acids, they tend to form closer contacts with R between 2.3 and 2.6 Å. As such, the protein-ligand SHBs are expected to exhibit considerable quantum effects in both the electronic and nuclear degrees of freedom.^6^ 81% of these SHBs have O as both the donor and acceptor atoms, while the rest form mostly between O and N atoms in the amino acid side chains and ligands (Table S3).

To evaluate the potential roles of protein-ligand SHBs in modulating the structures and functions of proteins, we group the ligands by their molecular geometries and chemical properties and identify a few key categories. As shown in Figure 1b, carbohydrates are the most abundant category and appear in 21.6% of protein-ligand SHBs. In particular, *α*-L-fucose, *β*-D-glucose, *α*-D-mannose and their derivatives are commonly observed to engage in SHB interactions with active-site residues of carbohydrate-binding proteins. As an example, Figure 1c shows the binding of 6-phosphonomethyl-6-deoxy-glucose (G7P) in the active site of *β*-phosphoglucomutase, forming a transition state analog along the isomerization pathway of converting *β*-D-glucose 1-phosphate to *β*-D-glucose 6-phosphate.^44^ The active-site residue Asp10 is positioned such that its carboxylate side chain hydrogen bonds with the 1-OH group of G7P, forming a close contact of 2.56 Å and possibly facilitating the general base catalysis of the enzyme.^44^ Nucleotides account for 21.4% of protein-ligand SHBs and 72% of them are pyridine nucleotides, i.e., nicotinamide adenine dinucleotide (NAD), nicotinamide adenine dinucleotide phosphate (NADP) and flavin nucleotides such as flavin mononucleotide (FMN) and flavin adenine dinucleotide (FAD). These nucleotide cofactors interact with dehydrogenases and flavoproteins and are essential electron carriers in cellular energy transfer and redox processes. For example, Figure 1d shows the active-site cavity of curacin cyclopropanase, an enoyl reductase that catalyzes the biosynthesis of cyclopropane in bacteria.^45^ The hydroxyl side chain of Ser163 forms a SHB with the phosphate group of NADP^+^ with an R of 2.64 Å, anchoring the cofactor for catalysis.^45^ From Figure 1b, acids and anions, such as fatty acids, citric acid and the malonate ion, are frequently observed in protein-ligand SHBs. Hemes are also commonly found to form these close contacts in a range of proteins including nitrophorin, myoglobin, cytochrome c and dehaloperoxidase-hemoglobin. In addition, non-proteinogenic amino acids such as S-adenosyl-L-homocysteine and D-glutamic acid are occasionally involved in the formation of SHBs. Given the large diversity of ligands, we group the remaining of them into the “Other” category. These include alcohol, drugs and metal-containing ligands and they make up for 33.6% of protein-ligand SHBs. For example, a variety of protease inhibitors, including indinavir, amprenavir and saquinavir, are observed to form SHBs with the catalytic Asp25 residue of HIV-1 protease, indicating the important role of these compact structures in the therapeutic treatment of the virus infection.^46–48^

### Chemical characteristics of protein-ligand SHBs

**Figure 2.**
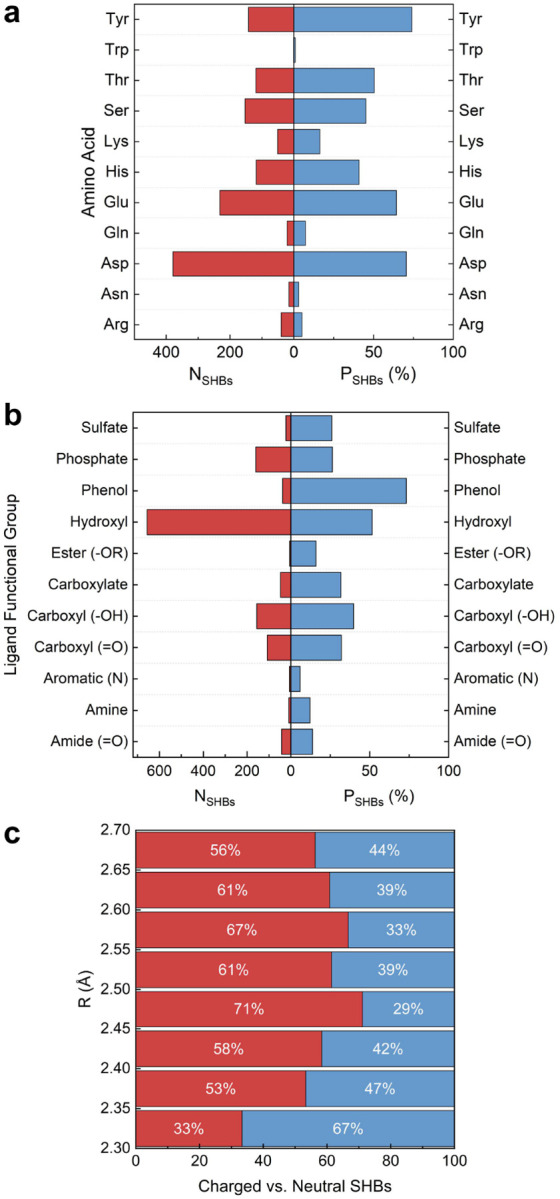
The number (*N*_*SHBs*_) and probability (*P*_*SHBs*_) of protein-ligand SHBs for (a) 11 amino acids with polar side chains and (b) representative functional groups in ligands. (c) Distribution of charged and neutral SHBs in the protein-ligand SHBs.

The protein alphabet contains 20 canonical amino acids, among which 11 have polar side chains and are capable of forming hydrogen bonds. As shown in Figure 2a, except Trp, all of them occur frequently in protein-ligand SHBs. Interestingly, the amino acids exhibit varying propensities to form SHBs, *P*_*SHBs*_, and can be categorized into three types: (A1) Tyr, Asp and Glu; (A2) Ser, Thr and His; (A3) Arg, Lys, Asn, Gln and Trp. Type A1 amino acids are highly probable to engage in SHB interactions. In particular, Tyr has a phenol side chain that forms 142 SHBs and 50 NHBs with ligands, showing the highest *P*_*SHBs*_ of 74% among all amino acids. This is followed by Asp and Glu with *P*_*SHBs*_ values of 71% and 64%, respectively. Their carboxylate side chains are strongly favored as acceptors in the SHBs, especially when the interacting heteroatoms are within a distance of 2.6 Å. In type A2, Ser and Thr possess hydroxyl groups while His has a neutral or cationic imidazole group in their side chains, and they collectively act as proton donors or acceptors in 390 protein-ligand SHBs. However, they are equally susceptible to participate in NHB interactions and their *P*_*SHBs*_ values are reduced to 45%, 50% and 41%, respectively. Unlike the other two cases, type A3 amino acids are more likely to form NHBs and their *P*_*SHB*_ are below 16%.

Compared to amino acids, ligands often have more complex molecular structures and comprise a variety of functional groups that can participate in hydrogen bonding interactions. For example, monosaccharides are aldehydes or ketones with multiple hydroxyl groups, heme contains a porphyrin ring that is often linked to carboxylate groups, and the pyridine nucleotides FAD and NADP are composed of nucleobases, riboses and phosphates. From Figure 2b, we identify 11 functional groups that are frequently observed in protein-ligand SHBs and categorize them into four types based on their respective *P*_*SHBs*_ values: (L1) phenol; (L2) alkyl hydroxyl; (L3) sulfate, phosphate, carboxyl and carboxylate; (L4) ester, amide, alkyl amine and N-containing aromatic heterocycle. Despite only participating in 52 hydrogen bonds, ligands containing phenol groups are highly prone to forming SHB interactions with amino acids. Specifically, 38 of these hydrogen bonds are SHBs, leading to a notable *P*_*SHBs*_ value of 73% for type L1 functional group. For type L2, alkyl hydroxyl groups form the largest number of protein-ligand hydrogen bonds and act primarily as the proton donors in 657 SHBs and 621 NHBs. Like the side chains of Ser and Thr, they are prone to form both classes of hydrogen bonds, resulting in a *P*_*SHBs*_ value of 51%. In contrast, functional groups in the other two types show significantly lower tendencies to participate in SHBs, with *P*_*SHBs*_ values ranging from 25% to 40% for type L3 and less than 14% for type L4. Figure 2a and b suggest that the presence of charges in the amino acid side chains and ligand functional groups could contribute to the formation of SHBs. As demonstrated in Figure 2c, we observe plentiful of SHBs with neutral donor and acceptor groups, but the majority of them contain at least one charged participant when R of the hydrogen bond is in the range of 2.35 – 2.7 Å.

### Development of the MAPSHB-Ligand model

Based on these analyses, we choose 14 input features for the development of the MAPSHB-Ligand model. These include the charge, residue type and heteroatom of an amino acid, and the charge and functional group of a ligand. We further use the acid and base dissociation constants (*pK*_*a*_ and *pK*_*b*_) and octanol-water partition coefficient (logP) to describe the ionization and lipophilicity characters of a ligand. In addition, we include the sequence of the neighboring 3 residues on both sides of the amino acid because the MAPSHB model has demonstrated that the adjacent protein sequence has a considerable influence on the propensity of an amino acid to form a SHB versus a NHB.^38^ The location of the amino acid in a protein-ligand hydrogen bond is fixed at the side chain of the protein.

**Figure 3.**
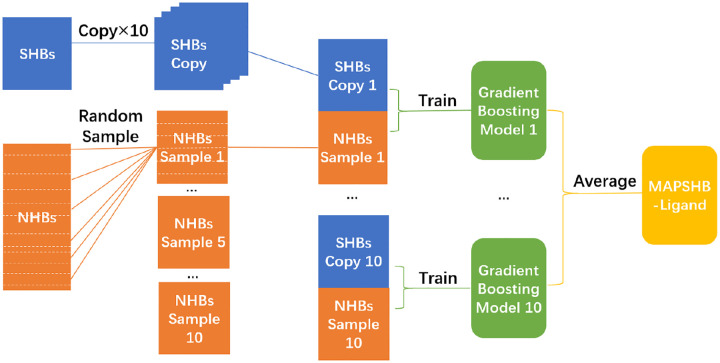
Schematic workflow for the development of the MAPSHB-Ligand model.

Given these chemical and sequence features, the MAPSHB-Ligand model is expected to attribute a protein-ligand hydrogen bond to a SHB or NHB. For this purpose, we randomly split the overall collection of hydrogen bonds with a 80:20 ratio and form a training set that contains 1019 SHBs and 2200 NHBs and a test set with 253 SHBs and 533 NHBs. Similar to protein-protein hydrogen bonds, the datasets of protein-ligand hydrogen bonds are imbalanced with twice as many NHBs as SHBs, and the classification prediction is strongly influenced by the inter-dependence among the 14 input parameters. We hence follow the procedures used for the MAPSHB model and invoke the undersampling and gradient boosting algorithms to construct the MAPSHB-Ligand model.^38^ As demonstrated in Figure 3, we start by randomly selecting 1019 NHBs from the training set and combining them with the SHBs to create a balanced dataset with an equal number of both classes of hydrogen bonds. This dataset is then used to train a gradient boosting model that employs a series of decision trees to account for the interaction effects of the 14 input features. Such an approach effectively captures the complex and non-linear relationships among the input features, resulting in accurate classification of hydrogen bonds into SHBs and NHBs.^49^ After repeating these steps on 10 different balanced datasets, we obtain a set of 10 gradient boosting models that collectively constitute the MAPSHB-Ligand model. We have implemented the model on our web server https://wanggroup.rutgers.edu/mapshb-model/the-mapshb-model. When a researcher submits a protein structure on the web server, the MAPSHB-Ligand model utilizes the 10 gradient boosting models to compute the probability that a protein-ligand hydrogen bond is a SHB, and outputs the averaged probability as the final result. The class of the hydrogen bond is then determined by comparing the predicted probability to a classification threshold: it is categories as a SHB if the probability is greater than or equal to the threshold, and as a NHB otherwise.

**Figure 4.**
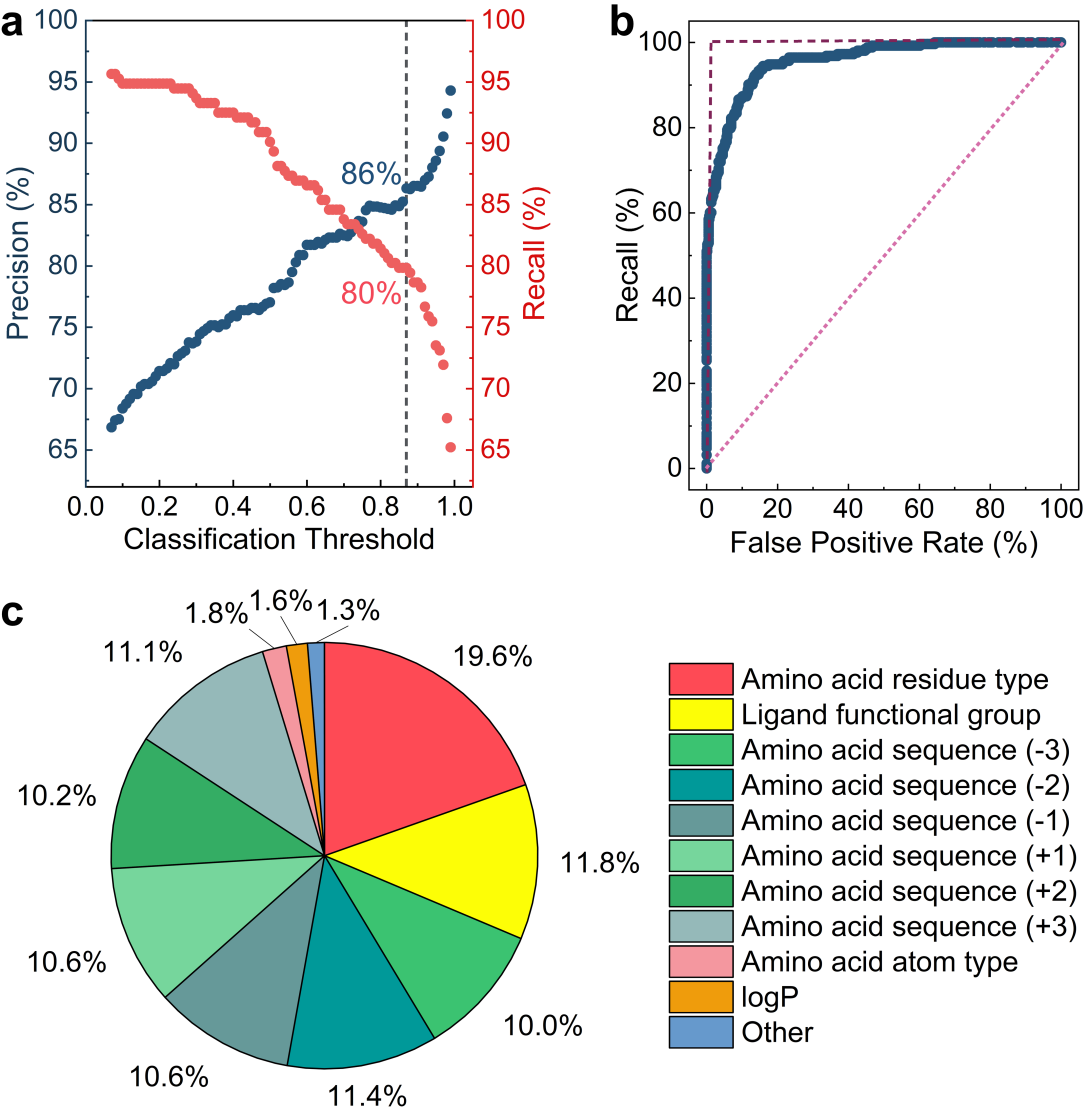
Analysis of the MAPSHB-Ligand model. (a) The precision and recall of the model as a function of the classification threshold. The vertical dashed line represents our recommended classification threshold of 0.870, which gives a 86% precision and 80% recall. (b) The ROC curve. The dashed vertical and horizontal lines represent the ROC curve of a perfect classification model, and the dotted diagonal line represents that of a random prediction model. (c) The normalized importance scores of the 14 input features. Features giving less than 1% contribution to the model prediction are combined into the “Other” category. These include the charge of the amino acid and the charge, *pK*_*a*_ and *pK*_*b*_ of the ligand.

To assess the effectiveness of the MAPSHB-Ligand model, we apply it to the test dataset and calculate two metrics, precision and recall, at various classification threshold values.^50^ Precision is computed as the fraction of real SHBs among the predicted SHBs, providing insights into the accuracy of the model in terms of true positive and false positive predictions. Recall is the percentage of correctly predicted SHBs within the total number of SHBs in the test dataset, and quantifies how complete the model can capture these short contacts in proteins. Both metrics are scaled between 0 and 100% and larger values indicate better model performance. As shown in Figure 4a, there is a clear trade-off between the two metrics and increasing the value of one comes at the expense of the other. By leveraging this property, one can adjust the classification threshold and tune the balance between precision and recall for a specific application. For example, when researchers aim to identify the vast majority of protein-ligand SHBs present in a protein structure, they could choose a small threshold of 0.062 to reach a recall rate of 96%, although the precision of the predictions would be limited to 67% (Table S7). On the other hand, if the primary objective is to precisely detect the occurrence of protein-ligand SHBs, researchers may opt for a high threshold of 0.996, which offers a precision of 98% despite of a lower recall rate of 56% (Table S7). We recommend using a classification threshold of 0.870, which is the same value chosen for the MAPSHB model. At this threshold, the MAPSHB-Ligand model achieves a 86% precision and 80% recall, demonstrating its ability to make accurate prediction of SHBs while identifying a substantial portion of them within a protein (Figure 4a).

Recognizing that precision and recall are computed at a single classification threshold, we further construct a receiver operating characteristic (ROC) curve and calculate the area under the curve (AUC) as a comprehensive metric to evaluate the model predictions across all possible classification thresholds.^51,52^ As demonstrated in Figure 4b, the ROC analysis plots the recall against the false positive rate of the binary classification, i.e., the fraction of NHBs that are erroneously classified as SHBs out of all NHBs in the test dataset, and each point arises from a different threshold value. The AUC as derived from an ROC curve ranges between 0 to 1, with a higher score suggesting a better performance of the model in separating the two classes of hydrogen bonds.^51,52^ For example, if a model randomly predicts the class of a hydrogen bond by flipping a fair coin, the ROC curve would follow the diagonal line where the recall equals the false positive rate, and the AUC score would be 0.5. In comparison, a perfect classification model accurately distinguishes between SHBs and NHBs and its ROC curve consists of two straight lines, as depicted in Figure 4b. It achieves an AUC score of 1, indicating flawless performance for both recall and false positive rate.^51^ From Figure 4b, the recall rate of the MAPSHB-Ligand model displays a rapid initial rise and approaches 100% as the false positive rate increases. Its ROC curve closely resembles that of a perfect model and the AUC score is 0.96, demonstrating the excellent ability of the MAPSHB-Ligand model to differentiate between SHBs and NHBs.

We then calculate the relative importance scores of the 14 input features and uncover three key factors that determine the prediction of the MAPSHB-Ligand model. As shown in Figure 4c, the residue type of amino acids and the functional group of ligands play the most important roles in the model predictions and their importance scores are 19.6% and 11.8%, respectively. Consistent with Figure 2a and b, the MAPSHB-Ligand model would provide a high SHB probability when the proton donor and acceptor groups consist of type A1 amino acids, such as Asp and Glu, and type L1 or L2 functional groups, such as the alkyl or aromatic hydroxyl groups of the ligands. Conversely, the model would produce a small SHB probability for the combination of type A3 amino acids and type L4 ligand groups, e.g., the amide side chain of Gln and the amine group of ligand. Note that hydrogen bonds that involve type A2 amino acids and type L2 or L3 ligand groups have a similar likelihood of forming either SHBs or NHBs. In such cases, the MAPSHB-Ligand model will consider additional input features such as the atom type of amino acids and logP of ligands to make a definite classification. From Figure 4c, although each individual residue surrounding a hydrogen bonded amino acid has a relatively low importance score (~10%), their collective impact on the model prediction is significant and the protein sequence plays a crucial role in modulating the formation of SHBs with ligands. For example, the Asp residues in the sequences of Gly-Ser-Glu-*Asp*-Gly-Thr-Asp and Asp-Gly-Thr-*Asp*-Asn-Asp-Tyr are often involved in SHB interactions with carbohydrates.^53–57^ In fact, these sequences are located in the calcium and monosaccharide binding loop of the lectin PA-IIL, and are conserved in various PA-IIL-like proteins found in bacteria.^55^ Apart from these three factors, the other input features combine to give an importance score of 4.7% (Figure 4c), suggesting that they have relatively minor influences on the model predictions.

As a summary, we have examined the top 1% highest quality structures in the PDB and developed the MAPSHB-Ligand model that effectively detects the presence of SHBs formed between amino acid side chains and small molecule ligands. We further integrate this model into a web server (https://wanggroup.rutgers.edu/mapshb-model/the-mapshb-model) and provide researchers with convenient access to analyze these specialized interactions. The combination of the MAPSHB-Ligand and MAPSHB models presents an efficient approach for investigating protein-protein and protein-ligand interactions involving SHBs, especially in cases where the protein structures are of moderate or low resolution. The predictions obtained from these models can serve as additional restraints in the experimental and computational refinement of protein structures, and aid in the elucidation of the structural basis of protein-protein and protein-ligand interactions. The machine learning models can be further refined and optimized with the continuous advancements in the filed of structural biology and the increasing availability of high-quality protein structures. These and other models will enable new engineering strategies to enhance the stability and functions of proteins and facilitate rational drug design efforts to achieve improved efficacy by leveraging SHB interactions as a key molecular mechanism.

## Computational methods

After collecting 1070 high-resolution structures from the PDB, we added H atoms to the amino acid residues and analyzed the protein-ligand complexes using the Amber 2016 software package.^58^ The ligand structures were determined from their Crystallographic Information Files (CIF). We then modeled the proteins and ligands using the Amber14SB force field^59,60^ and the general Amber force field (GAFF),^61^ respectively, and optimized the complexes while holding the non-H atoms at their positions in the crystal structures. We used three geometric criteria to identify a hydrogen bond: the heteroatoms are O or N atoms; 2.3 Å ≤ R ≤ 3.2 Å; the Donor-H-Acceptor angle ≥ 135°. For each hydrogen bond, we obtained the charge, residue and heteroatom of the amino acid, the charge of the ligand, and the relevant sequence information from the Amber 2016 program.^58^ The *pK*_*a*_, *pK*_*b*_ and logP of ligands were estimated using the Molecular Operating Environment (MOE) software v2020.0901.^62^ The functional groups of ligands were determined from their atomic connections.

We used the R programming language to develop the MAPSHB-Ligand model. A gradient boosting model was created for each balanced dataset by invoking the gbm function^63^ with an exponential loss function, using 5000 decision trees and a shrinkage of 0.01. We treated the interaction depth as a hyperparameter and determined it through 10-fold cross-validation. Specifically, we randomly divided each balanced dataset into 10 equal-sized subsets, from which 9 subsets were allocated for training the gradient boosting model and the remaining one was used for validation. This process was repeated 10 times with each subset serving as the validation set once. Each training-validation combination was referred to as a fold. In each fold, we trained the model using a candidate interaction depth between 1 and 12, evaluated its performance by applying it to the validation set, and recorded the loss. We then computed the final loss of the candidate interaction depth as the average of the losses obtained from all folds. We determined the optimal interaction depth as the one with the lowest final loss, and proceeded to retrain the gradient boosting model using the entire balanced dataset. The varImp function in the caret package^64^ was used to calculate the importance scores for each gradient boosting model, and the overall importance scores for the MAPSHB-Ligand model were obtained by averaging over those from the 10 boosting models. The data of the ROC curve were generated using the plotROC package,^65^ and the AUC score was computed using the auc function from the pROC package.^66^ More details of the hydrogen bond analysis and the development and evaluations of the machine learning models are provided in the Supplementary Information.

## Data Availability

All data generated or analysed during this study are included in this published article and its Supplementary Information file.

## References

[1] Baker E. N. & Hubbard R. E. Hydrogen bonding in globular proteins. Prog. Biophys. Mol. Biol. 44, 97–179 (1984). 6385134 10.1016/0079-6107(84)90007-5

[2] Flocco M. M. & Mowbray S. L. Strange bedfellows: Interactions between acidic side-chains in proteins. J. Mol. Biol. 254, 96–105 (1995). 7473763 10.1006/jmbi.1995.0602

[3] Rajagopal S. & Vishveshwara S. Short hydrogen bonds in proteins. FEBS J. 272, 1819–1832 (2005). 15819878 10.1111/j.1742-4658.2005.04604.x

[4] Panigrahi S. K. & Desiraju G. R. Strong and weak hydrogen bonds in the protein-ligand interface. Proteins: Struct., Funct., Bioinf. 67, 128–141 (2007). 10.1002/prot.21253 17206656

[5] Qi H. W. & Kulik H. J. Evaluating unexpectedly short non-covalent distances in x-ray crystal structures of proteins with electronic structure analysis. J. Chem. Inf. Model. 59, 2199–2211 (2019). 30875210 10.1021/acs.jcim.9b00144

[6] Zhou S. & Wang L. Unraveling the structural and chemical features of biological short hydrogen bonds. Chem. Sci. 10, 7734–7745 (2019). 31588321 10.1039/c9sc01496a PMC6764281

[7] Huggins M. L. 50 years of hydrogen bond theory. Angew. Chem. Int. Ed. 10, 147–152 (1971).

[8] Hibbert F. & Emsley J. Hydrogen bonding and chemical reactivity. Adv. Phys. Org. Chem. 26, 255–379 (1990).

[9] Perrin C. L. & Nielson J. B. “strong” hydrogen bonds in chemistry and biology. Annu. Rev. Phys. Chem. 48, 511–544 (1997). 9348662 10.1146/annurev.physchem.48.1.511

[10] Tuckerman M. E., Marx D., Klein M. L. & Parrinello M. On the quantum nature of the shared proton in hydrogen bonds. Science 275, 817–820 (1997). 9012345 10.1126/science.275.5301.817

[11] Steiner T. The hydrogen bond in the solid state. Angew. Chem. Int. Ed. 41, 48–76 (2002). 10.1002/1521-3773(20020104)41:1<48::aid-anie48>3.0.co;2-u 12491444

[12] Raugei S. & Klein M. L. Nuclear quantum effects and hydrogen bonding in liquids. J. Am. Chem. Soc. 125, 8992–8993 (2003). 15369337 10.1021/ja0351995

[13] Grabowski S. J. What is the covalency of hydrogen bonding? Chem. Rev. 111, 2597–2625 (2011). 21322583 10.1021/cr800346f

[14] Li X.-Z., Walker B. & Michaelides A. Quantum nature of the hydrogen bond. Proc. Natl. Acad. Sci. USA 108, 6369–6373 (2011).

[15] Ceriotti M. Nuclear quantum effects in water and aqueous systems: Experiment, theory, and current challenges. Chem. Rev. 116, 7529–7550 (2016). 27049513 10.1021/acs.chemrev.5b00674

[16] Dereka B. Crossover from hydrogen to chemical bonding. Science 371, 160–164 (2021). 33414217 10.1126/science.abe1951

[17] Cleland W. W. Low-barrier hydrogen bonds and low fractionation factor bases in enzymic reactions. Biochemistry 31, 317–319 (1992). 1731889 10.1021/bi00117a001

[18] Frey P., Whitt S. & Tobin J. A low-barrier hydrogen bond in the catalytic triad of serine proteases. Science 264, 1927–1930 (1994). 7661899 10.1126/science.7661899

[19] Cleland W. & Kreevoy M. Low-barrier hydrogen bonds and enzymic catalysis. Science 264, 1887–1890 (1994). 8009219 10.1126/science.8009219

[20] Cleland W. W., Frey P. A. & Gerlt J. A. The low barrier hydrogen bond in enzymatic catalysis. J. Biol. Chem. 273, 25529–25532 (1998). 9748211 10.1074/jbc.273.40.25529

[21] Mildvan A. Short, strong hydrogen bonds on enzymes: NMR and mechanistic studies. J. Mol. Struct. 615, 163–175 (2002).

[22] Yamaguchi S. Low-barrier hydrogen bond in photoactive yellow protein. Proc. Natl. Acad. Sci. U.S.A. 106, 440–444 (2009). 19122140 10.1073/pnas.0811882106 PMC2626721

[23] Dajnowicz S. Direct visualization of critical hydrogen atoms in a pyridoxal 5′-phosphate enzyme. Nat. Commun. 8, 955 (2017). 29038582 10.1038/s41467-017-01060-y PMC5643538

[24] Agback P. & Agback T. Direct evidence of a low barrier hydrogen bond in the catalytic triad of a serine protease. Sci. Rep. 8, 10078 (2018). 29973622 10.1038/s41598-018-28441-7 PMC6031666

[25] Kumar P., Serpersu E. H. & Cuneo M. J. A low-barrier hydrogen bond mediates antibiotic resistance in a noncanonical catalytic triad. Sci. Adv. 4, eaas8667 (2018). 10.1126/sciadv.aas8667 PMC5884680 29632894

[26] Kumar P. Low-barrier and canonical hydrogen bonds modulate activity and specificity of a catalytic triad. Angew. Chem. Int. Ed. 58, 16260–16266 (2019). 10.1002/anie.201908535 31515870

[27] Dai S. Low-barrier hydrogen bonds in enzyme cooperativity. Nature 573, 609–613 (2019). 31534226 10.1038/s41586-019-1581-9

[28] Kemp M. T., Lewandowski E. M. & Chen Y. Low barrier hydrogen bonds in protein structure and function. Biochim. Biophys. Acta Proteins Proteom. 1869, 140557 (2021). 33148530 10.1016/j.bbapap.2020.140557 PMC7736181

[29] Drago V. N. An n···h···n low-barrier hydrogen bond preorganizes the catalytic site of aspartate aminotransferase to facilitate the second half-reaction. Chem. Sci. 13, 10057–10065 (2022). 36128223 10.1039/d2sc02285k PMC9430417

[30] Warshel A., Papazyan A. & Kollman P. On low-barrier hydrogen bonds and enzyme catalysis. Science 269, 102–106 (1995). 7661987 10.1126/science.7661987

[31] Ash E. L., Sudmeier J. L., De Fabo E. C. & Bachovchin W. W. A low-barrier hydrogen bond in the catalytic triad of serine proteases? theory versus experiment. Science 278, 1128 (1997). 9353195 10.1126/science.278.5340.1128

[32] Schutz C. N. & Warshel A. The low barrier hydrogen bond (lbhb) proposal revisited: The case of the asp-his pair in serine proteases. Proteins 55, 711–723 (2004). 15103633 10.1002/prot.20096

[33] Fuhrmann C. N., Daugherty M. D. & Agard D. A. Subangstrom crystallography reveals that short ionic hydrogen bonds, and not a his-asp low-barrier hydrogen bond, stabilize the transition state in serine protease catalysis. J. Am. Chem. Soc. 128, 9086–9102 (2006). 16834383 10.1021/ja057721o

[34] Perrin C. L. Are short, low-barrier hydrogen bonds unusually strong? Acc. Chem. Res. 43, 1550–1557 (2010). 20939528 10.1021/ar100097j

[35] Oltrogge L. M. & Boxer S. G. Short hydrogen bonds and proton delocalization in green fluorescent protein. ACS Cent. Sci. 1, 148–156 (2015). 27162964 10.1021/acscentsci.5b00160 PMC4827562

[36] Lin C.-Y. & Boxer S. G. Unusual spectroscopic and electric field sensitivity of chromophores with short hydrogen bonds: Gfp and pyp as model systems. J. Phys. Chem. B 124, 9513–9525 (2020). 33073990 10.1021/acs.jpcb.0c07730 PMC8549526

[37] Pinney M. Structural coupling throughout the active site hydrogen bond networks of ketosteroid isomerase and photoactive yellow protein. J. Am. Chem. Soc. 140, 9827–9843 (2018). 29990421 10.1021/jacs.8b01596

[38] Zhou S., Liu Y., Wang S. & Wang L. Effective prediction of short hydrogen bonds in proteins via machine learning method. Sci. Rep. 12, 469 (2022). 35013487 10.1038/s41598-021-04306-4 PMC8748993

[39] Zhou S. & Wang L. Short hydrogen bonds in proteins. In Reference Module in Chemistry, Molecular Sciences and Chemical Engineering (Elsevier, 2022).

[40] Gippert G. P., Yip P. F., Wright P. E. & Case D. A. Computational methods for determining protein structures from NMR data. Biochem. Pharmacol. 40, 15–22 (1990). 2196882 10.1016/0006-2952(90)90172-h

[41] Feig M. Computational protein structure refinement: almost there, yet still so far to go. WIREs Comput. Mol. Sci. 7, e1307 (2017). 10.1002/wcms.1307 PMC6319934 30613211

[42] Moriarty N. W. Improved chemistry restraints for crystallographic refinement by integrating the amber force field into *phenix*. Acta Crystallogr. D 76, 51–62 (2020). 10.1107/S2059798319015134 PMC6939439 31909743

[43] Berman H. M. The protein data bank. Nucleic Acids Res. 28, 235–242 (2000). 10592235 10.1093/nar/28.1.235 PMC102472

[44] Jin Y. α-fluorophosphonates reveal how a phosphomutase conserves transition state conformation over hexose recognition in its two-step reaction. Proc. Natl. Acad. Sci. USA 111, 12384–12389 (2014). 25104750 10.1073/pnas.1402850111 PMC4151737

[45] Khare D. Structural basis for cyclopropanation by a unique enoyl-acyl carrier protein reductase. Structure 23, 2213–2223 (2015). 26526850 10.1016/j.str.2015.09.013 PMC4670573

[46] Liu F. Kinetic, stability, and structural changes in high-resolution crystal structures of hiv-1 protease with drug-resistant mutations l24i, i50v, and g73s. J. Mol. Biol. 354, 789–800 (2005). 16277992 10.1016/j.jmb.2005.09.095 PMC1403828

[47] Shen C.-H., Wang Y.-F., Kovalevsky A. Y., Harrison R. W. & Weber I. T. Amprenavir complexes with hiv-1 protease and its drug-resistant mutants altering hydrophobic clusters. FEBS J. 277, 3699–3714 (2010). 20695887 10.1111/j.1742-4658.2010.07771.x PMC2975871

[48] Olajuyigbe F., Demitri N. & Geremia S. Investigation of 2-fold disorder of inhibitors and relative potency by crystallizations of hiv-1 protease in ritonavir and saquinavir mixtures. Cryst. Growth Des. 11, 4378–4385 (2011).

[49] Friedman J. H. Greedy function approximation: A gradient boosting machine. Ann. Stat. 29, 1189–1232 (2001).

[50] Powers D. Evaluation: From precision, recall and f-measure to roc, informedness, markedness & correlation. J. Mach. Learn. Technol. 2, 37–63 (2011).

[51] Hanley J. A. & McNeil B. J. The meaning and use of the area under a receiver operating characteristic (roc) curve. Radiology 143, 29–36 (1982). 7063747 10.1148/radiology.143.1.7063747

[52] Hajian-Tilaki K. Receiver operating characteristic (roc) curve analysis for medical diagnostic test evaluation. Casp. J. Intern. Med. 4, 627 (2013). PMC3755824 24009950

[53] Sudakevitz D. A new ralstonia solanacearum high-affinity mannose-binding lectin rs-iil structurally resembling the pseudomonas aeruginosa fucose-specific lectin pa-iil. Mol. Microbiol. 52, 691–700 (2004). 15101976 10.1111/j.1365-2958.2004.04020.x

[54] Perret S. Structural basis for the interaction between human milk oligosaccharides and the bacterial lectin PA-IIL of Pseudomonas aeruginosa. Biochem. J. 389, 325–332 (2005). 15790314 10.1042/BJ20050079 PMC1175109

[55] Mitchell E. P. High affinity fucose binding of pseudomonas aeruginosa lectin pa-iil: 1.0 å resolution crystal structure of the complex combined with thermodynamics and computational chemistry approaches. Proteins: Struct., Funct., Bioinf. 58, 735–746 (2005). 10.1002/prot.20330 15573375

[56] Pokorná M. Unusual entropy-driven affinity of chromobacterium violaceum lectin cv-iil toward fucose and mannose,.Biochemistry 45, 7501–7510 (2006). 16768446 10.1021/bi060214e

[57] Marotte K. X-ray structures and thermodynamics of the interaction of pa-iil from pseudomonas aeruginosa with disaccharide derivatives. ChemMedChem 2, 1328–1338 (2007). 17623286 10.1002/cmdc.200700100

[58] Case D. AMBER 2016 (University of California, San Francisco, 2016).

[59] Ponder J. W. & Case D. A. Force fields for protein simulations. Adv. Protein Chem. 66, 27–85 (2003). 14631816 10.1016/s0065-3233(03)66002-x

[60] Maier J. A. ff14SB: Improving the accuracy of protein side chain and backbone parameters from ff99SB. J. Chem. Theory Comput. 11, 3696–3713 (2015). 26574453 10.1021/acs.jctc.5b00255 PMC4821407

[61] Wang J., Wolf R. M., Caldwell J. W., Kollman P. A. & Case D. A. Development and testing of a general amber force field. J. Comput. Chem. 25, 1157–1174 (2004). 15116359 10.1002/jcc.20035

[62] Molecular Operating Environment (MOE) (Chemical Computing Group ULC, 1010 Sherbooke St. West, Suite #910, Montreal, QC, Canada, H3A 2R7, 2020).

[63] Greenwell B., Boehmke B., Cunningham J. & GBM Developers. gbm: Generalized Boosted Regression Models (2020). R package version 2.1.8.

[64] Kuhn M. caret: Classification and Regression Training (2020). R package version 6.0–86.

[65] Sachs M. C. plotroc: A tool for plotting roc curves. J. Stat. Softw. 79, 1–19 (2017). 30686944 10.18637/jss.v079.c02 PMC6347406

[66] Robin X. proc: an open-source package for r and s+ to analyze and compare roc curves. BMC Bioinf. 12, 77 (2011). 10.1186/1471-2105-12-77 PMC3068975 21414208

